# Recurrent Herpes Simplex Virus 2 Lymphocytic Meningitis in Patient with IgG Subclass 2 Deficiency

**DOI:** 10.3201/eid2604.190406

**Published:** 2020-04

**Authors:** Tanvi Goyal, Imran Ali

**Affiliations:** University of Toledo, Toledo, Ohio, USA

**Keywords:** herpes simplex virus, HSV-2, meningitis, recurrent benign lymphocytic meningitis, RBLM, IgG2 immunodeficiency, viruses

## Abstract

We report a case of a patient with a lifetime history of 8 episodes of recurrent lymphocytic meningitis. Our findings suggest that susceptibility to recurrent lymphocytic meningitis might be caused by low serum IgG subclass 2 immunodeficiency.

Recurrent benign lymphocytic meningitis (RBLM) is an uncommon cause of meningitis; its clinical features include episodes of aseptic meningitis followed by complete recovery and unpredictable recurrence (*1*). The most common cause is herpes simplex virus-2 (HSV-2), a member of the family *Herpesviridae*. According to older case series, HSV has accounted for an estimated 0.5%–18% of viral meningitis cases (*2*). A study conducted by Kallio-Laine et al. (*1*) in Finland among 665 patients treated for lymphocytic meningitis indicated a prevalence of HSV-2–associated meningitis of 2.2 cases/100,000 population. RBLM is estimated to occur after primary HSV-2 meningitis in 20%–30% of cases (*3*). Previous research has reported an association of low serum IgG subclass 1 (IgG1) and IgG subclass 3 (IgG3) levels with increased frequency of RBLM (*2*). We report an unusual case of serum IgG subclass 2 immunodeficiency in a patient with 8 lifetime episodes of RBLM. 

## The Case

The case-patient was a 61-year-old woman with a history of RBLM. She had experienced 7 episodes of viral meningitis during 1978–1998. Her eighth episode occurred >20 years later. During this latest episode, the patient sought care for acute onset of bilateral diffuse headaches, with pain radiating to the neck. Additional symptoms included nausea and photophobia.

Physical examination revealed a temperature of 98.2°F, nuchal rigidity, positive Kernig sign, and no rash. Serum laboratory results showed neutrophilic and lymphocytic leukocytosis (15% neutrophils and 66% lymphocytes, reference value 0 for both). The total nucleated cell count was 283 cells/μL (reference range 0–5 cells/μL). Cranial computed tomography without contrast was unremarkable. Cerebrospinal fluid (CSF) analysis showed pleocytosis with a lymphocytic predominance, elevated protein at 114 mg/dL (reference range 15–45 mg/dL), and normal glucose at 44 mg/dL (reference range 40–70 mg/dL), compared with a serum glucose level of 83 mg/dL. A CSF culture with Gram stain did not show bacterial growth.

The patient also was tested for autoimmune diseases and potential immunodeficiency to determine a possible cause for her recurrent meningitis. Antinuclear antibody was positive with a titer of 1:160; however, the extractable nuclear antigen panel was negative. Chromatin IgG, double-stranded DNA, Smith, and RNA antibodies were all negative by ELISA. Immunoglobulin testing showed decreased IgM at 26 mg/dL (reference range 45–281 mg/dL), indicating an IgM deficiency. IgG subclasses showed decreased IgG2 at 130.3 mg/dL (reference range 242–700 mg/dL) and normal (reference) levels of IgG1, IgG3, and IgG4.

The patient received 1 dose intravenously of each of the following: vancomycin (20 mg/kg), ceftriaxone (2 g), and ampicillin (2 g) until the CSF meningitis panel showed HSV-2, at which time the patient’s treatment was switched to intravenous acyclovir (10 mg/kg) every 8 hours for 3 days. She was discharged on day 3, noting improvements in headache and neck pain, with a 7-day course of oral valacyclovir (500 mg) twice daily. Further review showed no history of oral or genital herpes infection.

## Conclusions

The syndrome of idiopathic recurrent lymphocytic meningitis was first described in 1944 by French neurologist Pierre Mollaret for 3 patients who had short-lasting episodes of recurrent fever, headache, and vomiting caused by aseptic meningitis (*4*). Although the term “Mollaret’s meningitis” is reserved for idiopathic recurrent aseptic meningitis (a term established before the advent of PCR), and recurrent meningitis attributable to HSV-2 (or less commonly, Epstein–Barr virus) is called “recurrent viral meningitis,” the 2 conditions are clinically indistinguishable (*4**,**5*).

More than 50% of patients with recurrent lymphocytic meningitis attributable to HSV-2 do not report a history of genital herpes (*5*). Moreover, active genital vesicular lesions during episodes of viral HSV-2 meningitis are not a consistent finding (*5*).

Why some patients with HSV-2 infection have recurrent viral meningitis is not known. However, some evidence points toward an immune-mediated pathology in RBLM. Franzen-Röhl et al. (*6*) showed that, contrary to their hypothesis, patients with recurrent HSV-2 meningitis have a stronger HSV-specific cell-mediated immune response compared with patients with recurrent genital HSV-2 infections and healthy seropositive persons. RBLM patients were found to have increased expression of Toll-like receptors (TLRs) and increased interferon (IFN)-α and specific T cell responses, suggesting that the pro-inflammatory state leads to a more severe clinical disease because of destruction of the blood–brain barrier, tissue remodeling, and vascular leakage (*6*). A case report by Willmann et al. (*7*) demonstrated a TLR-3 deficiency in a patient with RBLM and called for testing of TLR-3 alleles to gain further information on this receptor’s relevance to RBLM. Bonnin et al. (*8*) reported a case of RBLM associated with a complement factor 1 deficiency, and Snowden et al. (*9*) reported a case of RBLM associated with hereditary isolated IgG3 deficiency.

IgG1 and IgG3, which largely act against protein components such as tetanus and diphtheria toxin, mediate antibody-dependent cellular cytotoxicity important for clearance of bacterial and viral infections. IgG2 forms immune complexes to trigger complement-independent macrophage and polymorphonuclear cell–mediated phagocytic activity largely against polysaccharide capsules of certain bacteria, including *Streptococcus pneumonia*, *Neisseria meningitides*, and *Haemophilus influenzae* (*10*). Antiviral IgG can prevent infection, decrease virus replication, clear viral infection, and eliminate or lessen the severity of disease (*11*).

IFNs induce antiviral activity in many cell types. The ability of IFN-γ to inhibit replication of herpes simplex–1 viruses in mouse macrophages correlated with the cells’ production of nitric oxide (*12*). Inoue et al. (*13*) reported that interferon-γ production by peripheral blood mononuclear cells was decreased among patients with IgG2 deficiency. Furthermore, Kondo et al. (*14*) reported that reduced expression of IFN-γ messenger RNA might play a role in IgG2 deficiency. This research points toward a mechanism by which IgG2 deficiency might lead to recurrent viral meningitis episodes and the associated pathology.

In a study of 21 patients with RBLM, Kallio-Laine et al. (*2*) showed that low serum IgG1 levels were associated with increased frequency of recurrent meningitis episodes. They also reported a trend toward lower serum IgG3 in RBLM patients. However, to our knowledge, no previous research has specifically linked low levels of serum IgG2 with increased frequency of recurrent HSV-2 meningitis episodes.

In addition, although single Ig deficiencies on their own might not necessarily increase susceptibility to infection, combined influences of multiple deficiencies might modulate disease progression and be clinically relevant. Our findings demonstrate a possible immune linkage with increased frequency of RBLM, but more research is needed to determine how this information can be used in the management and treatment of RBLM patients.

Clinicians should suspect HSV-2 in all cases of RBLM, given that HSV-2 is the most common cause. Recognition of RBLM will lead to earlier diagnoses, decreased hospital stays, less unnecessary testing, better clinical treatment, and improved outcomes. Whereas previous research and case reports have shown an association of RBLM with deficiencies in IgG1 and IgG3, our findings suggest that susceptibility to RBLM might also be caused by low serum immunoglobulin IgG2 (*2**,**9*). Although some research has already suggested an immunologic pathology predisposing to recurrent HSV-2 viral meningitis, more research is needed to understand the complete underlying pathophysiology and determine how this information can be used to evaluate, manage, and improve outcomes for patients with RBLM.
